# Dual-energy CT parameters in correlation to MRI-based apparent diffusion coefficient: evaluation in rectal cancer after radiochemotherapy

**DOI:** 10.1177/2058460120945316

**Published:** 2020-09-17

**Authors:** Andreas P Sauter, Antonia Kössinger, Stefanie Beck, Dominik Deniffel, Hendrik Dapper, Stephanie E Combs, Ernst J Rummeny, Daniela Pfeiffer

**Affiliations:** 1 Department of Diagnostic and Interventional Radiology, School of Medicine, Technical University of Munich, Klinikum rechts der Isar, Munich, Germany; 2 Department of Radiation Oncology, School of Medicine, Technical University of Munich, Klinikum rechts der Isar, Munich, Germany; 3 Department of Radiation Sciences (DRS), Institute of Radiation Medicine (IRM), Helmholtz Zentrum München, Neuherberg, Germany; 4 Deutsches Konsortium für Translationale Krebsforschung (dktk), Partner Site Munich, Munich, Germany

**Keywords:** Rectal neoplasms, X-ray computed tomography, magnetic resonance imaging, contrast media, diffusion magnetic resonance imaging

## Abstract

**Background:**

Rectal cancer (RC) is a frequent malignancy for which magnetic resonance imaging (MRI) is the most common and accurate imaging. Iodine concentration (IC) can be quantified with spectral dual-layer computed tomography CT (DL-CT), which could improve imaging of RC, especially for evaluation of response to radiochemotherapy (RCT).

**Purpose:**

To compare a DL-CT system to MRI as the non-invasive imaging gold standard for imaging of RC to evaluate the possibility of a response evaluation with DL-CT.

**Material and Methods:**

Eleven patients who received DL-CT as well as MRI before and after RCT of RC were retrospectively included into this study. For each examination, a region of interest (ROI) was placed within the tumor. For MRI, the mean apparent diffusion coefficient (ADC) was assessed. For DL-CT, IC, z-effective, and Hounsfield Units (HU) were measured. IC, z-effective, and HU were normalized to the aorta. ADC was correlated to absolute and relative normalized IC, z-effective, and HU with Spearman’s ρ. Differences before and after treatment were tested with Wilcoxon signed-rank test.

**Results:**

HU, IC, and Z-effective values in DL-CT images decreased significantly after RCT (*P*<0.01 for each comparison). The mean ADC increased significantly after RCT. Spearman’s ρ of the absolute IC difference and the absolute ADC (both before and after RCT) is high and significant (ρ = 0.73; *P* = 0.01), whereas the ρ-value for z-effective (ρ = 0.56) or HU (ρ = 0.45) to ADC was lower and non-significant.

**Conclusion:**

Response evaluation of RC after RCT could be possible with DL-CT via the measurement of IC.

## Introduction

Rectal cancer (RC) is a frequent malignancy and represents the second most common malignancy of the large intestine (1,2). Compared to other subsides of the colon, younger patients are affected with an overall five-year survival rate of 66.5% (3). For determination of the local tumor stage (T-stadium), transrectal endoscopic ultrasound is the most accurate modality. However, this is an invasive examination with possible complications and imaging of local lymph nodes is limited. Magnetic resonance imaging (MRI) is the most sensitive non-invasive imaging modality regarding tumor infiltration and local lymph nodes (4). Computed tomography (CT) is an essential imaging modality in oncological diseases in general. In RC, it is used for staging purposes regarding distant metastases, especially in lung, liver, and distant lymph nodes or within the peritoneum (5). Neoadjuvant radiochemotherapy (RCT) is the standard of care for locally advanced tumors (6). Evaluation of treatment response can be performed accurately with MRI – particularly with diffusion-weighted imaging (DWI) and the resulting apparent diffusion coefficient (ADC) (4,7,8). Hereby, differences between ADC values obtained with 1.5-T and 3-T systems seem to be small with discrepancies of less than 5% (9). However, contraindications for MRI exist, such as severe claustrophobia and metal implants, e.g. non-MRI-safe pacemakers or valve implants (10).

CT can be used for imaging of the primary tumor; however, therapy response to RCT can only be visualized indirectly by size measurement as the tumor perfusion and thus its vitality cannot be quantified in single-phase examinations with conventional CT systems (11,12). This drawback of CT could be overcome with dual-energy CT (DE-CT) systems, which have found widespread application in clinical routine in recent years (13). In contrast to conventional CT systems, with these systems, spectral data can be obtained. Different approaches for acquisition of spectral data exist, such as dual-source CT (DS-CT), rapid kVp-switching CT, kVp-switching with artificial intelligence reconstruction, or dual-layer CT (DL-CT) (14–17). DL-CT uses one constant tube voltage and spectral data are realized by a detector with a top layer of a yttrium-based garnet scintillator which detects low-energy photons and a bottom layer of gadolinium oxysulphide which detects high-energy photons (18). Using these data, low- and high-energy images are obtained and by weighted summation, conventional images and spectral data can be calculated. Spectral information can be used for multiple applications (13,19–21). One of those is the differentiation and quantification of materials such as iodine and thus imaging of the perfusion becomes feasible (22,23). Via perfusion imaging, blood volume can be estimated via the amount of iodinated contrast medium present, visualizing microvascular function and thus indicating tumor hypoxia and angiogenesis (12). Hereby, a normalization to the aorta or a large artery can be performed to minimize influences of the patient’s individual circulation via dividing the iodine concentration (IC) of the lesion by the IC of a large artery (e.g. the external iliac artery or the aorta) (24,25). With DL-CT systems, spectral data are acquired in every scan and thus a full retrospective spectral evaluation of CT data is possible. Previous studies showed that even small ICs can be measured accurately with DL-CT (23,26,27). Via detection and quantification of iodine, evaluation of lesions and masses becomes possible, such as differentiation of pulmonary metastases from different primary tumors or evaluation of complex cystic renal masses (28,29). During the last years, multiple approaches were made for therapy assessment using DE-CT (20,30–32). For example, response evaluation in patients with malignant melanoma or gastrointestinal stroma tumors was shown to be possible (30,31). Despite the additional spectral information, DL-CT is not associated with an increased radiation dose (33,34).

Until now, response evaluation of RC after RCT with CT is only possible by size measurements. With DE-CT systems, information regarding tumor vitality could become measurable via quantification of iodine uptake which represents the perfusion of the tumor (35,36). To the best of our knowledge, there are no studies comparing MRI-based ADC and spectral information from DE-CT. However, studies evaluating MRI-based ADC in RC exist, enabling a comparison of DE-CT and these existing ADC-values (37–41).

In the present study, MRI as the non-invasive imaging gold standard was compared to a DL-CT system to evaluate the possibility of a response evaluation with DL-CT.

## Material and Methods

### Approval

This retrospective study was approved by the local ethics committee. Thus, no additional data or examinations were acquired for the study. All data were completely anonymized at the beginning of the study. Informed consent was waived by the institutional review board due to the retrospective design.

### Study cohort (patient population)

**Fig. 1.**
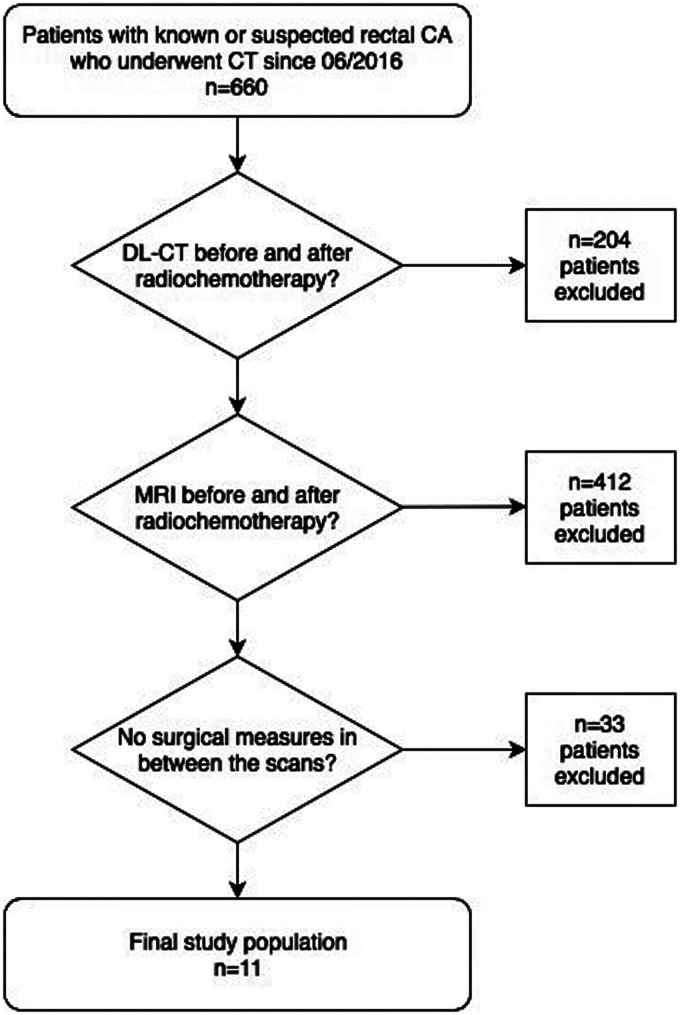
Flow chart showing selection and exclusion criteria to receive the final study population of 11 patients.

The hospital information system (Picture Archiving and Communication System [PACS]) was searched for patients diagnosed with RC who underwent neoadjuvant RCT since September 2016 (n = 660). The search yielded 11 patients with a diagnosis of rectal carcinoma who were treated with RCT and had comparable DL-CT and MRI scans before and after the therapy (Fig. 1).

Patients were excluded for the following reasons, in this order: (i) a DL-CT scan either before or after the RCT was not available (n = 204); (ii) lack of an MRI scan either before or after the treatment (n = 412); and (iii) patients who underwent RCT but also received surgery between CT or MRI scans (n = 33).

Patients received RCT with a total of 50.4 Gy (1.8 Gy per radiation) and 5-FU intravenously (week 1 and 5) or Cepecitabine orally (at each radiation) (42,43).

### Dual-energy CT technique

The examination of all patients using a dual-layer spectral CT (IQon; Philips Healthcare, The Netherlands) followed the same routine protocol. Before the start of the scan, an anteroposterior scout was performed to determine the scan range. Iodinated intravenous contrast medium (Imeron 400 MCT, 400 mg/mL; Bracco Imaging Deutschland GmbH, Konstanz, Germany) was injected with a standard dosage of 1.2 mL/kg body weight at a flow rate of 2–2.5 mL/s, followed by a 30-mL saline chaser at the same flow rate. All scans were performed in the venous phase (scan delay time amounted to 70 s after the start of contrast medium).

All scans were performed using a collimation setting of 64 × 0.625 mm, a tube voltage of 120 kVp, and an automatic exposure control. The field of view was adapted to the patient size. For all scans, an image matrix of 512 × 512 was used. All images were reconstructed with slice thickness and interval of 0.9 mm/0.9 mm with a soft-tissue kernel; with these images, greater slice thicknesses and multiplanar reconstructions can be generated at each workstation. Conventional and spectral basis images were reconstructed using the iDose4 (Philips Healthcare, The Netherlands) algorithm.

### Magnetic resonance imaging

CT and MRI scans before therapy were acquired within a median of two days (mean = 2.9 days, range = 0–17 days). After therapy, the scans were acquired within a median of one day (mean = 1.2 days, range = 0–6 days; two outliers with 195/337 days were not included in this calculation).

MRI was performed on one 1.5-T System (Magnetom Avanto, Siemens Medical Solutions, Erlangen, Germany) and on three 3-T Systems (Verio and Biograph mMR, Siemens Medical Solutions, Erlangen, Germany and Ingenia, Philips Healthcare, Best, The Netherlands). The diffusion-weight images were obtained using an echo planar imaging sequence with tri-directional diffusion gradients. Eight examinations were performed using the 1.5-T system, the remaining examinations were performed using a 3-T system.

Table 1 presents the parameters that were used in the 1.5-T and 3.0-T MRI systems.

**Table 1.** Parameters of DWI for the 1.5-T and 3.0-T MR systems used.

	1.5 T	3.0 T
b-values (mm^2^/s)	50, 300, 500	50, 300–400, 600–1000
Echo time (ms)	76	61–75
Section thickness (mm)	5	2.5–5
iPAT factor	2	2
Distance factor (%)	20	40
FOV (mm)	300	281–300
Repetition time (ms)	4700	10,344–11,401
EPI factor	154	49–102

DWI, diffusion-weighted imaging; FOV, field of view.

### Image analysis

The image analysis was performed using the commercially available software solution Philips IntelliSpace Portal (2018) (Version 10.1.0.21400) by two radiologists (with 6 and 10 years of experience in MRI, respectively) with the support of a medical student.

The scans before and after RCT of all 11 patients were analyzed following the same protocol.

For image analysis of the DL-CT scans, a circular region of interest (ROI) was placed in the tumor and the abdominal aorta. The ROIs were chosen to be as large as possible but without the risk of including areas with partial volume effects. The mean size of all ROIs was 24 mm^2^.

**Fig. 2.**
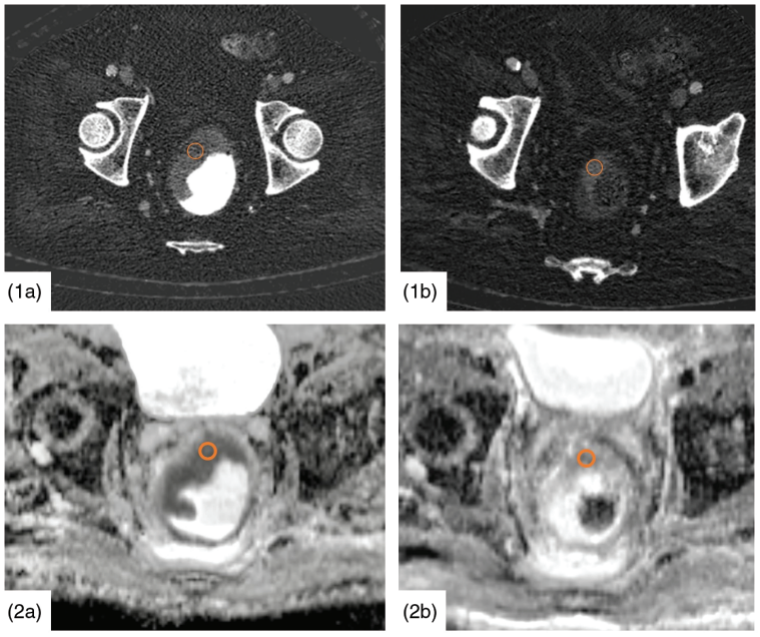
Dual-layer CT iodine imaging (1) of the tumor and MRI (2) before (1a, 2a) and after (1b, 2b) RCT. IC and ADC are measured via corresponding ROIs in CT and MRI images. The tumor stadium was rated uT3 (before RCT) and yT2 (after RCT), respectively. ADC, apparent diffusion coefficient; CT, computed tomography; IC, iodine concentration; MRI, magnetic resonance imaging; RCT, radiochemotherapy; ROI, region of interest.

The ROIs before and after the RCT were placed in the corresponding region of the tumor, depending on the tumor size (Fig. 2).

The mean value of the measured Hounsfield unit values (HU ROI), IC values (IC ROI), and Z-effective values (Z ROI) of the tumor were normalized with the mean value in the aorta (aorta reference value [ARV]) of 100 healthy patients who underwent DL-CT in our institution as a reference and the corresponding aortic values of the individual patient (aorta individual value [AIV]).

|(IC ROI)*(IC ARV)|/(IC AIV)

|(Z ROI Patient)*(Z ARV)/|(Z AIV)

|HU ROI​Patient)*(HU​ARV)/(HU AIV)|

For the MRI analysis, the ADC was measured via a circular ROI which was placed into the tumor in the corresponding MRI scans before and after the therapy.

### Statistical analysis

Data were tested for gaussian distribution with the Anderson-Darling test. As a gaussian distribution was not present for any group, the Wilcoxon signed-rank test was conducted to evaluate the differences before and after RCT. A difference was considered statistically significant at *P* < 0.05. Correlation was tested with Spearman’s ρ. Hereby, a ρ value of 0.0–0.3 indicates negligible, 0.3–0.4 low, 0.5–0.7 moderate, 0.7–0.9 high, and 0.9–1.0 very high correlation given a significant *P* value (44). Regression analysis was performed using linear regression with least squares regression without weighting.

Statistical analyses were performed with SPSS Statistics (Version 25, SPSS Inc., Chicago, IL, USA) and GraphPad Prism (Version 8, San Diego, CA, USA)

## Results

### Study cohort

The final study cohort included 11 patients (six men, five women; mean age = 63.8 ± 11.2 years). The patients’ mean body mass index was 22.98 ± 2.86 kg/m^2^. The initial TNM tumor stages were uT2/uT3 cN+/cN1 M0 G2. The final tumor stages were ypT2/3 ypN0/1a L0/1 R0. Using the cut-off ADC value of 1200 mm^2^/s, 8/11 patients in the present study showed a complete response. The tumor stage of the three patients showing no complete response according to ADC was ypT3 yN0 for each patient. The mean volume-weighted CT dose index (CTDI_vol_) and dose-length product (DLP) for the complete protocol (chest, abdomen, and pelvis) were 8.7 mGy and 526.6 mGy*cm, respectively. This corresponds to a mean effective dose of 7.1 mSv.

### DL-CT

The mean HU, IC as well as Z-effective values in DL-CT images decreased significantly after RCT (*P* < 0.01 for each comparison) as shown in Tables 2 and 3. The measured HU decreased from 77.46 HU to 55.83 HU after RCT. Z-effective decreased slightly from 7.94 to 7.72.

**Table 2.** HU, IC, Z-effective, and ADC values before and after radiochemotherapy.

	HU before	HU after	IC before (mg/mL)	IC after (mg/mL)	Z-eff before	Z-eff after	ADC before (mm^2^/s)	ADC after (mm^2^/s)
Minimum	61.27	38.08	1.24	0.43	7.36	6.96	533.0	882.0
Maximum	92.54	79.18	2.50	2.02	8.48	8.21	1216	1491
Range	31.27	41.10	1.26	1.59	1.12	1.25	683.0	609.0
Mean	77.46	55.83	1.74	1.00	7.94	7.72	926.2	1257
SD	10.91	11.75	0.44	0.49	0.36	0.40	175.5	210.3
SEM	3.29	3.54	0.13	0.15	0.11	0.12	52.92	63.40

ADC, apparent diffusion coefficient; HU, Hounsfield units; IC, iodine concentration; SD, standard deviation; SEM, standard error of the mean; Z-eff, Z-effective.

**Table 3.** Absolute and relative change of HU, IC, Z-effective, and ADC ± SD after RCT compared to the values before RCT.

	HU	IC (mg/mL)	Z-effective	ADC (mm²/s)
Difference absolute	–21.3 ± 14.0	–0.74 ± 0.40	–0.22 ± 0.23	331 ± 227
Difference relative (%)	61.6 ± 26.5	43.5 ± 26.6	2.8 ± 0.8	40.1 ± 35.6

ADC, apparent diffusion coefficient; HU, Hounsfield units; IC, iodine concentration.

**Fig. 3.**
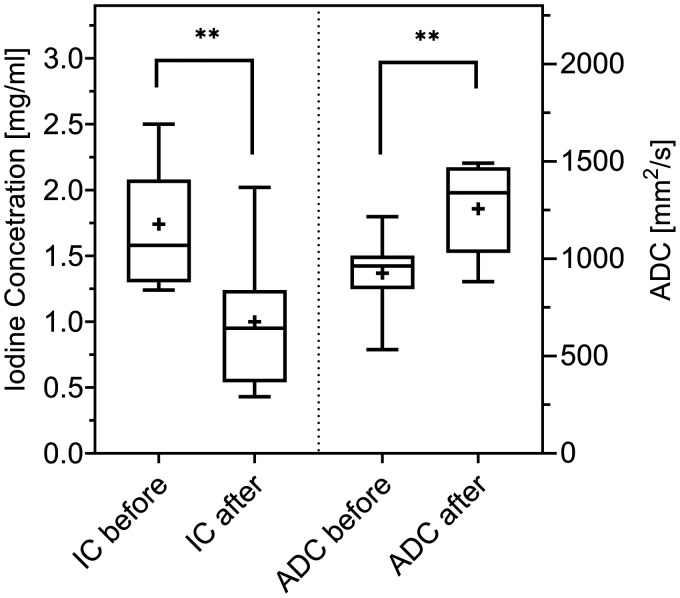
IC and ADC before and after RCT, shown as box and whisker plots. The horizontal line indicates the median, the cross indicates the mean, and the whiskers indicate the 5–95 percentile. **P < 0.01. ADC, apparent diffusion coefficient; IC, iodine concentration; RCT, radiochemotherapy.

**Fig. 4.**
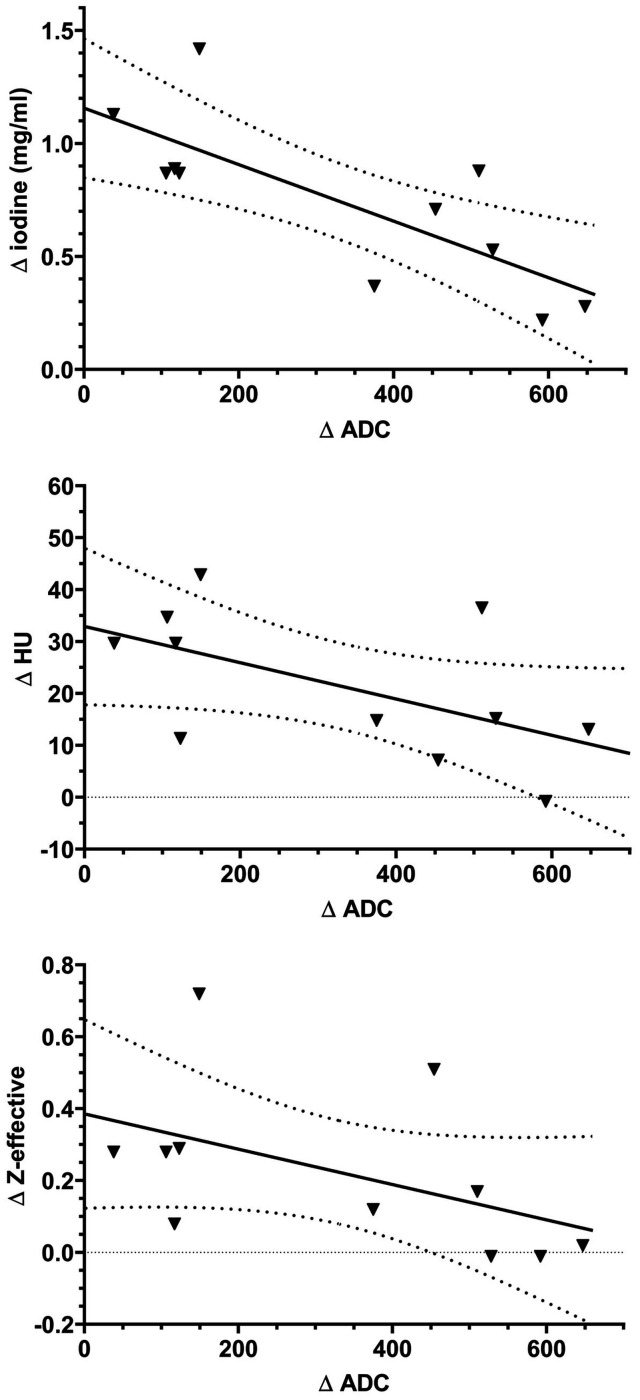
Regression analysis comparing IC, HU and z-effective to the ADC. The solid line shows the regression curve and the dotted line shows the 95% confidence interval. ADC, apparent diffusion coefficient; HU, Hounsfield units; IC, iodine concentration.

A comparison of the IC and ADC before and after RCT is shown in Fig. 3. Hereby, IC decreased from 1.74 mg/mL to 1.00 mg/dL. In the corresponding MRI images, ADC was significantly higher after RCT (1257 mm^2^/s) than before RCT (926.2 mm^2^/s, *P* < 0.01). The Spearman’s correlation coefficient of the absolute IC in DL-CT images and the absolute ADC difference in MRI (both before and after RCT) was high and significant (*r* = 0.73; *P* = 0.01), whereas the correlation of the absolute differences of ADC and HU (*r* = 0.45; *P* = 0.17) as well as of ADC and Z-effective (*r* = 0.56; *P* = 0.08) were not significant. A regression analysis for each comparison is shown in Fig. 4.

When relative differences before and after RCT were used, the Spearman’s ρ decreased for HU (ρ = 0.12; *P* = 0.735) and IC (ρ = 0.36; *P* = 0.285) but increased for Z-effective (ρ = 0.60; *P* = 0.054). In contrast to absolute values, the *P* value was not significant for any correlation.

## Discussion

DE-CT systems nowadays find widespread use and thus detection and quantification of iodine becomes possible in clinical routine. This advantage is already used in multiple settings such as the evaluation of renal masses (29). With iodine quantification, the iodine uptake can be evaluated in a single CT scan without the need of an unenhanced phase and so, the perfusion of tissues can be measured indirectly. As the perfusion of tumors such as rectal carcinoma decreases under successful therapy, response evaluation could be possible with DE-CT. For response evaluation of RC, MRI and especially the ADC value is the gold standard for non-invasive imaging (7). However, MRI is not always possible, for example for patients with pacemakers or valve implants. Additionally, CT is performed for staging purposes in every patient with advanced RC. Thus, an evaluation of the therapy response of the local tumor with CT in routine staging examinations seems reasonable, especially when an MRI examination is not available.

In the present study, ADC measured with MRI as the non-invasive gold standard was compared to IC, HU value, and Z-effective measured in a DL-CT to evaluate the possibility of a response evaluation of rectal carcinoma with DL-CT.

An excellent correlation of ADC and IC with ρ = 0.73 was shown for the examined patients. As the ADC increases with tumor response whereas the IC decreases, an inverse correlation was observed. For HU and Z-effective, lower and not significant Spearman’s ρ were found and thus IC seems the most promising parameter for evaluation of rectal carcinoma and the therapy response. We showed that when comparing ADC and IC, the absolute differences before and after RCT showed a higher ρ than the percentage changes of these values and that with percental differences, the correlation yielded non-significant ρ values. This is most likely due to the fact that there is no ground value for ADC and that relative calculations are therefore difficult. Thus, in the present study, absolute changes of IC are better applicable than relative changes. However, one has to keep in mind that absolute differences might be subject to greater differences than relative differences thus leading to wider confidence intervals.

Earlier studies indicated the possibility for response evaluation via ADC (7,45,46). These studies showed that a correlation between ADC value and tumor response exists. Different techniques for ADC evaluation were suggested as some studies stated a cut-off value of the absolute ADC for discrimination between complete response and not complete response and other studies suggested the usage of a relative ADC increase. All studies found ADC values in the same range as the present study. Using the cut-off ADC value of 1200 mm^2^/s as suggested in Kim et al. (7), 8/11 patients in the present study showed a complete response. However, due to the small patient collective, the recommendation for an IC cut-off cannot be made. The present study was designed to evaluate the correlation between ADC and IC, and as this correlation could be proven, further studies for the evaluation of a cut-off should be performed with a greater number of patients.

As earlier studies demonstrated that ICs < 1 mg/mL can be measured accurately, the iodine quantification in the current study with mean values before and after RCT of 1.74 and 1.00 mg/mL should be possible precisely (23,26). Thus, the evaluation of IC in rectal carcinoma is possible from a technical standpoint.

Despite the described benefits of DE-CT, there are some drawbacks that have to be addressed. The measurement could be impaired by metal artefacts, e.g. due to hip endoprosthesis or spinal fusion which must be considered during the evaluation; however, such artefacts were not present in this study. For all DE-CT systems except DL-CT, examinations have to be acquired accordingly to obtain spectral information and thus a retrospective analysis is not always possible. Furthermore, intravenous contrast medium can be contraindicated in patients with impaired renal function.

The present study has some limitations. First, only a small patient collective was examined. This is because for each patient, a DL-CT scan as well as an MRI had to be present before and after RCT. However, for this first feasibility study, the examined number of patients seems appropriate. Second, the present study only shows that a response evaluation of RC with DL-CT-based IC is possible with a high correlation to MRI. However, further studies must evaluate an IC cut-off in comparison to histological tumor staging after surgery to be able to differentiate therapy response from therapy failure and to determine how well the correlation of IC and tumor viability performs. Third, even MRI, which is widely accepted as the radiological gold standard for the assessment of RC, is inferior to endoscopic sonography in terms of diagnostic accuracy of assessment of tumor extend. However, MRI and CT are non-invasive and widely available and are thus excellent techniques for tumor staging. Fourth, no other DE-CT technology was used besides DL-CT as different systems are not available at our institution. As IC can be measured accurately with other systems (26), the results of the present study should be transferable to other DE-CT systems. Finally, circular ROIs were used instead of a smart ROI due to reproducibility and transferability between DL-CT and MRI. We believe that this is a common measurement method; however, even more accurate results could have been achieved using smart ROI.

In conclusion, the present study is the first to compare parameters from a DL-CT to ADC in MRI for the response evaluation of RC after RCT. An excellent correlation of IC and ADC could be shown and thus DL-CT could be the imaging modality of choice for evaluation of RC when MRI is contraindicated as well as in CT scans for tumor staging when an MRI examination is not (yet) available.
